# Long-term environmental unpredictability increases social information use in zebrafish

**DOI:** 10.1093/beheco/arag064

**Published:** 2026-06-10

**Authors:** Britney Sekulovski, Noam Miller

**Affiliations:** Department of Psychology, Wilfrid Laurier University, 75 University Ave. West, Waterloo, ONT, Canada, N2L 3C5; Department of Psychology, Wilfrid Laurier University, 75 University Ave. West, Waterloo, ONT, Canada, N2L 3C5

**Keywords:** zebrafish, social information, schooling, environmental unpredictability

## Abstract

Human-induced environmental change increases unpredictability, disrupting habitats and social structures in ways that many animals are poorly adapted to. This may also reduce the reliability of information faster as contingencies shift. While independently acquired information is often more accurate, increased unpredictability may make recency the key to reliability, increasing the value of social information, which is cheaper and faster to update. Unpredictability might thus shift the balance between the values of these types of information, increasing reliance on more recent social information. Thus, independent of information quality, the predictability of background conditions may affect how animals make decisions. To test whether living in an unstable environment changes the inherent value of information, zebrafish (*Danio rerio*) were housed for 3 mo under either highly unpredictable Dynamic (water temperature, feeding times, habitat complexity, group size and membership fluctuated) or Stable conditions. Across 6 behavioral assays, Dynamic condition fish showed more information-seeking, greater attention to social stimuli, more sensitivity to social cues, and were less coordinated but swam closer together in shoals. When personal and social information conflicted, they were also more likely to prioritize recent social information over previously learned personal information. Together, these results indicate that long-term housing in an unpredictable environment diminishes the value of information faster, raises the value of social information to allow for faster updating, shifts decision-making strategies independent of the quality of information itself, and disrupts coordinated schooling in zebrafish. This sensitivity may fundamentally alter how animal collectives navigate an increasingly unpredictable world.

## Teaser

We show that zebrafish that live in unpredictable conditions pay more attention to social information than fish from stable environments. This makes sense because, in unpredictable environments, information becomes outdated faster, and animals must spend more time gathering and updating it, which is costly. Information can come either from personal experience or from observing others, which is cheaper and might therefore be preferred under instability. Flexible information-gathering strategies may thus help animals survive in a rapidly changing world.

## Introduction

Natural environments differ in predictability, with some exhibiting consistent changes that animals can anticipate (eg, seasonal changes), while others experience abrupt stochastic perturbations, fluctuating unpredictably and making environmental cues less reliable predictors of future conditions (eg, anthropogenic change; Wingfield and Kitaysky 2002; Bernhardt et al. 2020; Sih 2024). Environmental variation may be temporal (changes over time in temperature, habitat complexity, resource availability, or social group structure) or spatial (differences across locations in habitat structure, resource distribution, predation pressure, or population density; Rotenberry and Wiens 1980; Wiens 1985).

With the accelerating pace of human-induced environmental changes, including habitat disruption and climate instability, animals are increasingly confronted with unpredictable conditions (Acevedo-Whitehouse and Duffus 2009; Domenici and Seebacher 2020; Sih 2024), which may require them to rapidly and flexibly adjust their behavior. Ectotherms are especially vulnerable to extreme thermal variability, which can impair their metabolism, immune function, growth, and overall fitness (Angilletta et al. 2002), with unpredictable changes constraining anticipatory physiological regulation (Wingfield and Kitaysky 2002). Rising water temperatures also disrupt ocean productivity, causing declines in fish populations (Lluch-Belda et al. 1989; Lluch-Cota 2000; Nevárez-Martínez et al. 2001; Lanz et al. 2009). In response to warming sea surface temperatures and the resulting reduced prey availability, many species of seabirds rapidly adjust their reproductive strategies by changing their breeding time (Dobson et al. 2017) or increasing the spacing of egg-laying (increasing the survival chances of the first hatchling, which may outcompete or eliminate its sibling if needed; Bizberg-Barraza et al. 2024). Urgent questions remain about how animals make decisions and adjust their behavior to adapt to unpredictable environments, defined by the lack of reliable anticipatory cues.

Environmental variability may reduce the reliability of all information as contingencies change more frequently (Riotte-Lambert and Matthiopoulos 2020). The value of information depends on how closely it matches current conditions, and therefore declines faster in more variable environments, which require increased information updating behavior (Boyd and Richerson 1988). For example, tropical agamid lizards (*Psammophilus dorsalis*) from more spatially and temporally variable environments outperform those from stable environments on learning and cognitive flexibility tasks, demonstrating a higher capacity to track and use new information (Batabyal and Thaker 2019). Similarly, in more variable environments, using the most recent information becomes more important, as older information is more likely to be outdated. For example, when payoffs change unpredictably, pigeons shift to using the most recent feedback for decision-making (Bell and Baum 2002). As the value of new information increases in variable environments, individuals may benefit from engaging in more information-seeking behaviors, such as environmental vigilance (scanning) and sampling (Dall et al. 2005; Dridi and Lehmann 2016).

There are two types of information that can be used to guide decision-making: personal and social. Personal information is gathered through direct interaction with the environment (Kendal et al. 2009). While often accurate and directly relevant to the individual, personal information can be costly to acquire, typically through trial-and-error, requiring significant time and energy, and entailing exposure to risks such as predation or competitive losses (Coolen and Giraldeau 2003; Kendal et al. 2009). In contrast, social information is acquired by observing others, offering a cheaper (in time and energy) and safer alternative (Laland 2004; Kendal et al. 2009). While the speed and low cost of acquisition makes social information easier to update, it can be misleading, outdated, or not relevant to the individual or current context (Kendal et al. 2009).

When possible, incorporating both types of information in decision-making has been shown to lead to more accurate decisions and is considered the optimal strategy (Valone 1989; Templeton and Giraldeau 1996; Valone and Templeton 2002). However, the two sources may contradict each other, indicating different behavioral responses, in which case information of each kind should be weighted depending on its relative reliability (Pérez-Escudero and de Polavieja 2011). For example, ninespine sticklebacks (*Pungitius pungitius*) tend to prefer their own personal information over conflicting social information (van Bergen et al. 2004), likely because personal information is typically more reliable and relevant to the individual (Kendal et al. 2009). However, sticklebacks do not show this preference if their personal information is unreliable or outdated, instead conforming to social information (van Bergen et al. 2004). A similar switch to relying on more recent social information has been observed across species when the quality of personal information decreases, and is sometimes referred to as a “copy when uncertain” strategy (Rafacz and Templeton 2003; Laland 2004; Smolla et al. 2016).

Animals’ environments may also be socially variable, such as in fission–fusion systems, where subgroup size and membership change on relatively short timescales (Croft et al. 2003). Primates from such variable-membership groups outperform those from less complex groups on multiple cognitive tasks involving learning, behavioral flexibility, and social cognition (Amici et al. 2008; Aureli et al. 2008; MacLean et al. 2012), possibly because frequent partner turnover increases the cognitive demands of constant updating of information about individuals and social dynamics (Amici et al. 2008). Due to the importance of social interaction to the survival of many species (Krause and Ruxton 2002), social instability may have an outsized effect on the evolution of strategies to manage unpredictability.

Increased environmental unpredictability may shift the optimal balance between personal and social information (Valone and Templeton 2002). Animals may shift to prioritizing social information in variable environments, as it is cheaper to obtain and faster to update (Kendal et al. 2009). However, social information is acquired secondhand and, as a result, might not have been updated by direct experience for some time (Boyd and Richerson 1988). Making good decisions therefore relies on a complex interaction between the recency of information, its source, and the variability of the environment. Here, we explore one part of this relationship, testing how living in highly predictable or unpredictable environments (both physically and socially) affects the relative weighting of personal and social information in zebrafish.

Zebrafish are highly gregarious, exhibiting a range of complex social behaviors (Miller and Gerlai 2011). In the wild, they form large shoals ranging from 5 to 300 (Pritchard et al. 2001; Spence et al. 2008). In still water, zebrafish form relatively stable groups with an average of 3.5 fission–fusion events per minute, whereas in fast-flowing water this increases to around 8 per minute (Shelton et al. 2020). Their collective motion spans the range from loose aggregations (shoals) to highly coordinated schools, which are faster and less dense (Miller and Gerlai 2012a). The physical habitats occupied by zebrafish often fluctuate in temperature, vegetation cover, food availability, and social composition (Engeszer et al. 2007; Lawrence 2007; Spence et al. 2008; Shelton et al. 2020), and they are classified as one of the most eurythermal fish species (see Lawrence 2007), tolerating temperatures from 6.7 to 41.7 °C (Cortemeglia and Beitinger 2005; Schaefer and Ryan 2006).

Their remarkable tolerance to variable conditions and demonstrated complex social behaviors make zebrafish well-suited for studying responses to environmentally induced phenotypic plasticity. Both physical and social housing conditions have been shown to affect individual and group behavior in zebrafish, even in adulthood (Shams et al. 2015; Dos Santos et al. 2020; Toni et al. 2025), consistent with their high levels of lifelong neuroplasticity and neurogenesis (Pushchina et al. 2024). For example, adult zebrafish housed at low temperatures (18 °C) show more anxiety-like behaviors, including more bottom-zone use and less exploration, whereas fish housed at high temperatures (34 °C) show increased shoal cohesion (Toni et al. 2025). Fluctuating thermal environments also have negative effects on development (Schaefer and Ryan 2006) and adult reproductive performance (Massey et al. 2022). However, the existing literature on these effects has focused largely on predictable environmental cycles, rather than on how long-term exposure to unpredictable background environmental change shapes behavior.

We housed zebrafish in either a Dynamic condition, where water temperatures and feeding times changed daily, and habitat complexity and group size and composition changed every third day, or in a Stable condition, where all variables were kept constant. It is important to note that our Stable condition is unusually consistent and not intended to represent a typical environment, as the natural habitats of zebrafish are quite variable. Rather, it provides a tightly controlled comparison to test how variation across physical and social factors influences behavior. Because prolonged stability may itself have behavioral effects, differences in behavior between the fish in our two conditions should be interpreted as consequences of differences in the relative predictability of environmental conditions.

Fish lived in these conditions for 3 mo and throughout testing, during which we assessed differences in exploratory behavior, social motivation, stress reactivity, group cohesion (closeness) and polarization (coordination), and decision-making strategies. We hypothesized that living in a dynamic environment increases the value of recent information, leading to more information updating and seeking behavior, and may also lead to increased reliance on social information. Based on this, Dynamic condition fish were expected to pay more attention to their surroundings, showing increased environmental sampling and social vigilance. Additionally, they should weight recent information more heavily when balancing conflicting information, ignoring older information independent of its quality.

## Methods

### Subjects and housing

Eighty 3-mo-old experimentally naïve wild-type zebrafish served as subjects (excluding stimulus and demonstrator fish). Fish were bred in-house under standard laboratory conditions (12:12 h light:dark) and housed in one of eight 38 L glass tanks (50.8 × 25.4 × 30.5 cm; 4 Stable, 4 Dynamic) containing reverse osmosis (RO) water with added salt (Instant Ocean), with 1 filter, heater, and air-stone per tank. All housing tanks also contained plastic plants glued to small rocks. Water quality was monitored daily (salinity 450 to 750 ppm TDS; pH 6.8 to 7.8). Fish were fed twice daily (Skretting GEMMA Micro plus brine shrimp; ∼0.10 g per 5 fish per feeding).

### Environmental manipulation

Dynamic condition tanks completed 5 consecutive 18-d cycles of variation before Post-exposure testing, and continued under the same regime during the testing. Environmental variation consisted of daily changes in temperature and feeding times, and changes every 3 d in group size and composition and habitat complexity (see Table S1 for details). Mean environmental parameters matched those of the Stable group.

Stable condition tanks were kept at 23 ± 1 °C, fed at 10:00 and 14:00 h every day, retained a fixed 4-plant layout, and always contained the same 10 fish. Handling matched the Dynamic condition, with brief bucket transfers on Switching days (fish from different tanks were placed into separate buckets). Every third Switching day (every ninth day), while fish were in buckets, all tanks received 25% water changes and tank/filter cleaning; on other Switching days, tanks were topped-up with RO water. Prior to housing assignment, fish were tested on 3 behavioral assays (Open-Field Test, Social Preference Test, and Novel Tank Diving Test; see below) to measure individual differences. Baseline scores were used to allocate fish to the two housing conditions such that mean values did not differ across conditions before exposure (see Results).

### Behavioral assays

Fish were tested on 6 behavioral assays: an open-field test (OFT), a social preference test (SPT), a novel tank diving test (NTDT), a schooling test, a social information test (SIT), and a conflicting information test (CIT). The OFT, SPT, and NTDT were conducted both before and after the 3-mo housing exposure; the other assays were only conducted post-exposure. Each assay is briefly described below, and detailed methods for all assays are available in the Supplementary Information. All testing was conducted at least 2 h after lights-on, to avoid potential confounding effects from spawning behaviors (zebrafish typically spawn within the first hour of illumination under laboratory conditions; Darrow and Harris 2004). Switching days were paused for assays that took over a day but continued otherwise. All tests were filmed to allow for subsequent coding of behaviors from the videos.

For the OFT (Figure S1), fish were placed alone in a novel tank with a shelter along one end; we measured the proportion of the session fish spent outside the shelter as a proxy for how exploratory they were (as in Guayasamin et al. 2017). For the SPT (Figure S1), fish were placed alone in a tank with 2 side chambers, one of which contained 5 novel stimulus fish; we quantified the proportion of the session that the test fish spent close (within 10 cm) to the chamber containing the other fish, indicating their social drive (Gonçalves et al. 2022). For the NTDT (Figure S2), commonly used as an assay for stress, fish were placed alone into a small, novel, brightly-lit tank and filmed from the side; stressed zebrafish will often respond by staying close to the bottom of the tank in this situation; we measured proportion of the session spent in the bottom third of the tank, time spent frozen, and number of crossings between vertical thirds of the tank (Kysil et al. 2017).

For the schooling test (Figure S3), groups of 5 fish from the same home tank were placed into a circular tank and allowed to swim for 10 min while being tracked; we measured the mean distance between all the fish (called the inter-individual distance, IID), the mean nearest-neighbor distance (NND), the polarization of the group (the degree to which they were swimming in the same direction), thigmotaxis (the tendency to stay close to the wall, often linked to stress levels; Scharf and Farji-Brener 2024), and the speed of the fish, all common measures of coordination and collective behavior (Miller and Gerlai 2012b). These measures were averaged over all fish in the group and over all frames in the video, and we also analyzed the 10 min session in 4 2.5 min segments.

For the SIT (Figure S4), fish were individually tested. Test fish were allowed to observe 3 demonstrator fish feeding on 10 floating food pellets at one of two feeders, and another 3 fish not feeding (at an empty feeder) on the other side of the tank. The demonstrators and any leftover pellets were then removed, the water between chambers was mixed, and the test fish was released into the empty arena to choose between the two sides; we quantified the number of pellets the demonstrators consumed (not always all of them), the test fish's first choice, and the proportion of the session the test fish spent on either side of the arena. This test is commonly used to explore the use of social information in the absence of any personal information (Coolen et al. 2003; van Bergen et al. 2004; Webster and Laland 2011). Finally, for the CIT, test fish were trained to locate food at one of the two feeders in the same tank as the SIT for 7 d (4 trials/day). Following this training, fish observed demonstrators feeding at the opposite feeder to the one they had been trained on (ie, the demonstrators provided social information that conflicted with the test fish's personal information), and the test fish were then released to select a feeder; we measured the test fish's initial choice, latency to choose a side, and time spent on either side of the arena (van Bergen et al. 2004). This test quantifies conformity (choosing based on social information in the presence of contradictory personal information).

### Analysis

Because we were unable to track individual identities across assays, results were analyzed at the group level, by housing condition (Stable or Dynamic). Additionally, because fish in the Dynamic condition were repeatedly mixed across tanks (part of the unpredictability of their environment), we could not consider different tanks as replicates, and we therefore did not include replicate as a random factor in the analyses.

After measures from all 6 assays were derived, they were each analyzed separately using JASP (JASP Team 2025). Housing conditions were compared with each other using ANOVAs where there were more than 2 scores to compare (such as in the NTDT, which was run both before and after the housing manipulation), and *t*-tests and Anderson–Darling tests (when the data were not normally distributed) for pairwise comparisons; continuous measures were analyzed using linear regressions. In addition to test statistics and *P*-values, we also report means ± SD for all measures (Table 1). For SIT and CIT, both categorical (first choice; χ^2^) and continuous measures (time proportions; *t*-tests) are reported, as it has not yet been established which behavioral measures best capture different forms of information use in this species. All analyses are reported to ensure transparency and to provide a foundation for refining behavioral metrics in future studies of information use in zebrafish. Sexes were unknown (zebrafish are not highly sexually dimorphic), so sex differences were not examined, though sex-based differences in behavior have been reported (Genario et al. 2020).

**Table 1:** Mean values ± SD of key measures for all behavioral assays.

Assay	Stable		Dynamic	
	Pre	Post	Pre	Post
OFT	0.41 ± 0.21	0.66 ± 0.18	0.42 ± 0.22	0.61 ± 0.22
SPT	0.84 ± 0.18	0.72 ± 0.21	0.82 ± 0.16	0.80 ± 0.23
NTDT (Bottom)	0.12 ± 0.19	0.11 ± 0.13	0.10 ± 0.22	0.07 ± 0.08
NTDT (Freezing)	0.13 ± 0.15	0.03 ± 0.13	0.16 ± 0.18	0.02 ± 0.07
NTDT (Zones)	118.38 ± 96.71	101.23 ± 65.70	120.10 ± 74.53	136.23 ± 68.15
Schooling (IID)	…	13.53 ± 5.08	…	9.77 ± 4.19
Schooling (NND)	…	6.45 ± 2.39	…	4.52 ± 1.48
Schooling (Pol)	…	0.57 ± 0.09	…	0.53 ± 0.10
Schooling (Speed)	…	1.09 ± 0.22	…	0.93 ± 0.39
Schooling (Thig)	…	0.806 ± 0.016	…	0.806 ± 0.017
SIT	…	0.59 ± 0.34	…	0.49 ± 0.4
CIT (Conform)	…	0.45 ± 0.18	…	0.74 ± 0.19
CIT (Conform SL)	…	0.43 ± 0.16	…	0.87 ± 0.23

OFT, Open-Field Test, values give the mean proportion of the session spent outside the shelter; SPT, Social Preference Test, values give the proportion of the session spent close to conspecifics; NTDT, Novel Tank Diving Test, values show the proportion of the session spent in the bottom third of the tank (Bottom) or frozen in place (Freezing), and the number of zone switches (Zones); Schooling, schooling test, values show the mean inter-individual distance (IID, in cm), nearest-neighbor distance (NND, in cm), group polarization (Pol), speed (Speed, in cm/s), and proportion of time close to the arena walls (Thig); SIT, Social Information Test, values show proportion of fish choosing the demonstrated feeder; CIT, Conflicting Information Test, values show proportion of all fish conforming to social information (Conform) or only “successful learners”, fish that passed Probe 1 (Conform SL).

In the SIT, trials were excluded if fish failed to leave the start box (*n* = 2 Dynamic), procedural errors occurred (*n* = 3 Dynamic; *n* = 2 Stable), or < 3 s were spent outside the neutral zone (*n* = 1 Stable); several fish died before completing this assay. A total of 61 fish (Stable, *n* = 36; Dynamic, *n* = 25) successfully completed the test. Time spent in all zones (including each chamber, the feeder zones in each chamber, and the neutral zone) was calculated, and preference scores were calculated as the proportion of time spent on the social information side, relative to the total time spent on both sides. In the CIT, trials were also excluded if fish failed to leave the start box (*n* = 2 Dynamic) or spent < 3 s out of the neutral zone (*n* = 4 Stable, 4 Dynamic). Additionally, more fish had died before trials for this assay started. A total of 50 fish (Stable, *n* = 31; Dynamic, *n* = 19) successfully completed the test.

Raw data from all the assays are available in our OSF repository (https://osf.io/286md/overview?view_only=952c73f5e14c4e5a8ba4eef6bee0fffc).

## Results

### Open-field test

**Figure 1:**
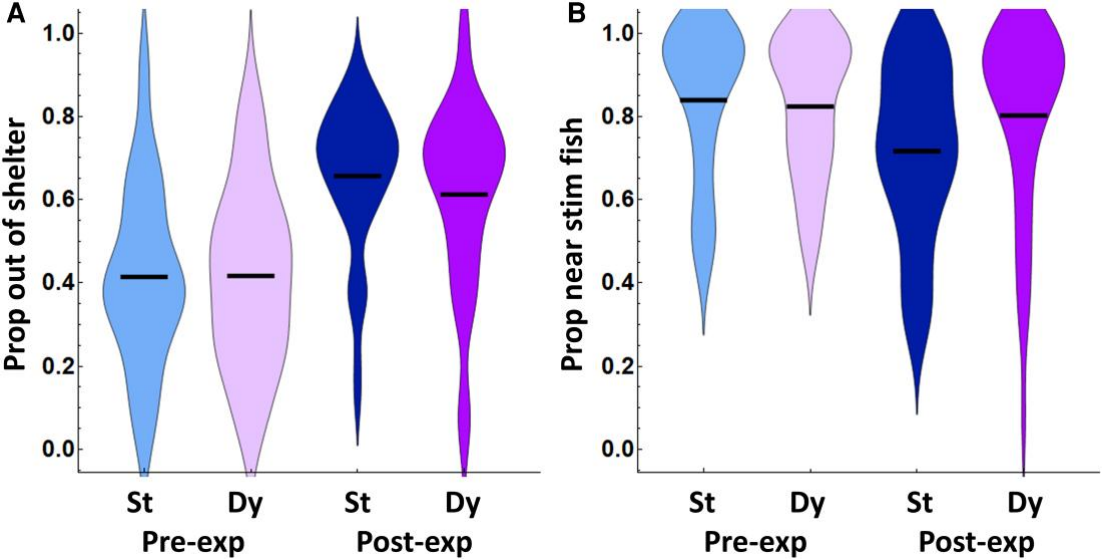
Effects of housing condition on exploratory behavior and social motivation. Violin plots of, a) proportion of time spent outside the shelter in the Open-Field Test and, b) proportion of time spent near the social stimulus in the Social-Preference Test for both Stable (“St”) and Dynamic (“Dy”) condition fish, before (“Pre-exp”) and after (“Post-exp”) a 3-mo exposure to the housing conditions. Short horizontal black lines indicate group means.

Exploratory behavior scores in the open-field test (OFT) were measured as the proportion of time each fish spent outside the shelter. As intended, Pre-exposure scores did not differ significantly between fish that would eventually be assigned to different conditions (Fig. 1a; Table 1; Anderson–Darling Test: *A* = 0.41, *P* = 0.83). After the 3-mo exposure period, exploratory behavior increased significantly in both groups (Stable: *A* = 11.99, *P* < 0.0001; Dynamic: *A* = 7.60, *P* = 0.0002). There was no significant difference between conditions (*A* = 0.75, *P* = 0.52), indicating that the increase in exploration was due to general habituation and/or ontogenetic effects rather than differential effects of the treatment.

### Social preference test

Social motivation in the SPT was assessed as the proportion of time each fish spent near the compartment containing a conspecific shoal. Pre-exposure scores did not differ significantly between housing conditions (Fig. 1b; Table 1; *A* = 0.36, *P* = 0.87), confirming that groups were behaviorally matched at baseline. Following the 3-mo exposure period, shoaling preferences declined in the Stable condition (from 0.84 to 0.72 [proportion time spent close to the social stimulus]; *A* = 5.16, *P* = 0.002), but fish in the Dynamic condition showed no significant change (0.82 Pre to 0.80 Post-exposure; *A* = 0.80, *P* = 0.48). Stable condition Post-exposure scores were significantly lower than the corresponding Dynamic scores (*A* = 3.21, *P* = 0.022).

### Novel-tank diving test (NTDT)

Pre-exposure scores showed no significant differences between the Stable and Dynamic groups across any of the measures (Figure S5; Table 1; time spent in bottom third: *t*(78) = −0.36, *P* = 0.721; zone crossings: *t*(78) = 0.089, *P* = 0.929; freezing duration: *t*(78) = 0.76, *P* = 0.450). These results confirm that behavioral stress levels were equivalent across conditions prior to the experimental manipulation.

Fish in both conditions exhibited slightly reduced bottom-dwelling Post-exposure, though this change was only significant in the Stable condition fish (Figure S5a; Stable: *A* = 2.69, *P* = 0.04; Dynamic: *A* = 1.86, *P* = 0.11; note that even though these tests compare the same fish before and after exposure, they are independent-sample tests, as individual identities could not be established). There was no difference between the conditions in Post-exposure time spent in the bottom third (*A* = 2.05, *P* = 0.09). As previous studies have assessed stress using shorter NTDT durations (Levin et al. 2007; Egan et al. 2009), we also analyzed time spent in the bottom third during only the first half of the trial (5 min). Results were consistent with the full-trial data (Figure S5b): fish in both conditions spent less time in the bottom-zone following exposure than before, though this effect was not significant in either condition (Stable: *A* = 1.16, *P* = 0.47; Dynamic: *A* = 0.81, *P* = 0.47), and there were no significant differences between conditions (*A* = 1.59, *P* = 0.16).

Freezing behavior also decreased significantly from Pre to Post-exposure in both groups (Figure S5c; Stable: *A* = 41.07, *P* < 0.0001; Dynamic: *A* = 37.32, *P* < 0.0001), and also showed no difference between the groups (*A* = 0.17, *P* = 0.99). The reduction in both bottom-dwelling and freezing is consistent with habituation to the novel environment, as we also observed in the OFT, and may also reflect ontogenetic changes over the experimental period. Vertical zone switches Post-exposure revealed a significant effect of housing conditions (Figure S5d). Dynamic condition fish switched zones more often than Stable condition fish (Table 1; *A* = 2.68, *P* = 0.040). Switching was not significantly different from Pre to Post-exposure in either condition (Dynamic: *A* = 1.25, *P* = 0.25; Stable: *A* = 0.49, *P* = 0.76).

### Schooling test

**Figure 2:**
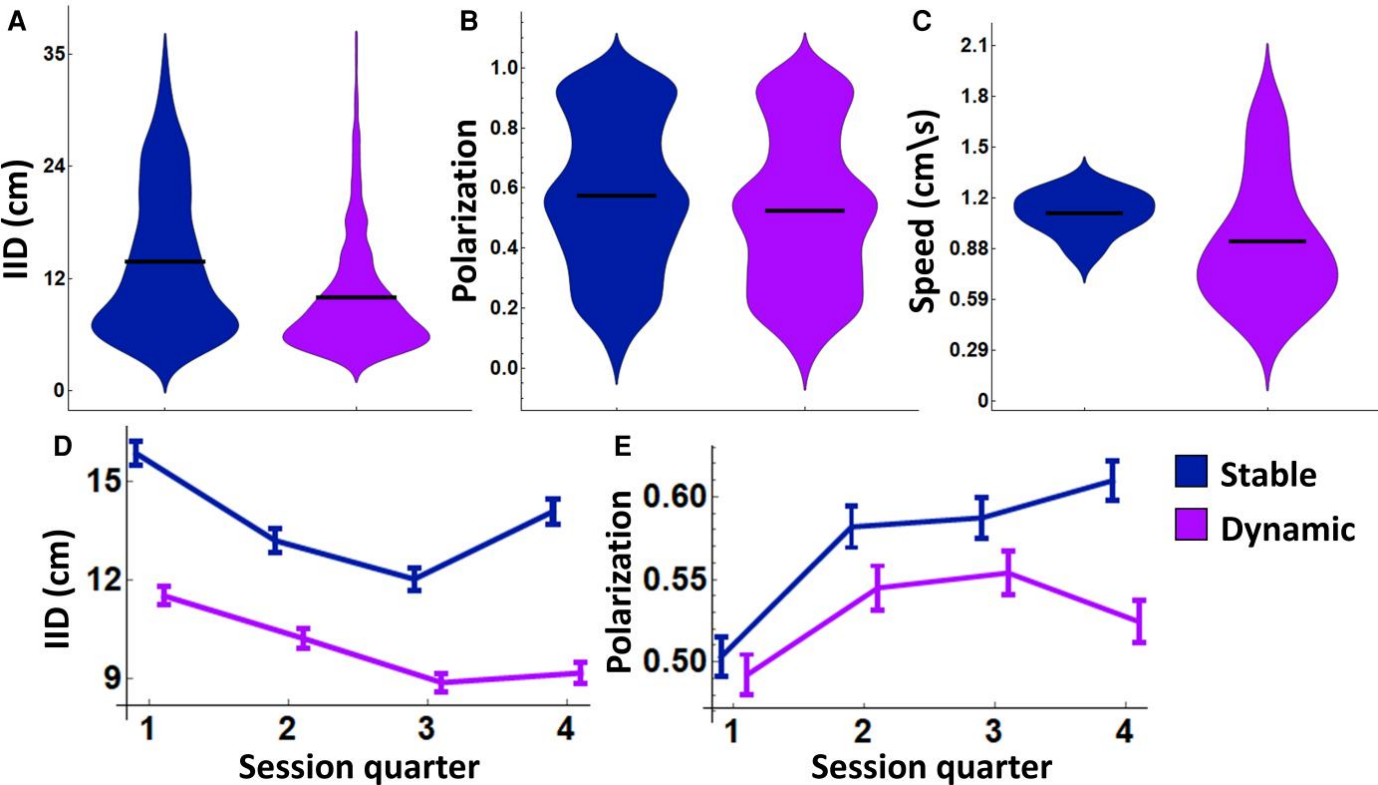
Effects of housing condition on schooling behavior. a) violin plot of the mean inter-individual distance (IID, in cm); b) violin plot of the mean polarization of the group; c) violin plot of the mean speed of the shoal (in cm/s); d) plot of mean IID for each quarter of the session; e) plot of mean polarization across quarters of the session. Short horizontal black lines in a–c show group means; error bars in d) and e) show ± SEM.

Stable condition fish formed looser shoals, maintaining larger distances between the fish (Fig. 2a; Table 1; IID: *A* = 12.12, *P* < 0.0001; NND: *A* = 15.58, *P* < 0.0001), were more polarized (Fig. 2b; *A* = 6.60, *P* = 0.0005), and swam faster (Fig. 2c; *A* = 2.67, *P* = 0.04) than Dynamic condition fish. The polarization of Dynamic condition shoals was significantly more variable (F-test of equality of variances: F = 0.32, *P* < 0.0001). Thigmotaxis, defined as the tendency to stay within the outer 10% of the tank radius, did not differ between conditions (Stable = 0.806 ± 0.016; Dynamic = 0.806 ± 0.017; *A* = 0.66, *P* = 0.59).

The characteristics of shoaling cohesion also varied across the 10 min sessions and differed across conditions. There was a significant interaction between condition and session segment for both IID (Fig. 2d; 2-way ANOVA: main effect of Condition, F(1, 3704) = 273.36, *P* < 0.0001; main effect of Segment, F(3, 3704) = 33.27, *P* < 0.00001; Condition × Segment interaction, F(3, 3704) = 4.056, *P* = 0.007; post-hoc tests showed all segments differed except S2 and S4) and polarization (Fig. 2e; Main effect of Condition, F(1, 3256) = 22.04, *P* < 0.0001; main effect of Segment, F(3, 3256) = 15.41, *P* < 0.0001; Condition × Segment interaction, F(3, 3256) = 3.13, *P* = 0.02; post-hoc tests showed segment S1 differed from the other 3).

### Social information

To measure social information use, fish first observed demonstrators feeding at one of two feeders on opposite sides of the tank, and were then allowed to choose between the feeders, in the absence of the demonstrators. We measured demonstrator performance (number of pellets consumed), the first chamber the test fish entered after release (first choice), and the time the test fish spent in both feeder zones. Mean feeder preference scores did not significantly differ between conditions (Table 1; *t*(59) = 1.03, *P* = 0.308). There was also no effect of condition on initial side choice (χ^2^ = 0.12, *P* = 0.730); 57.4% of fish chose the social information side first and first choice strongly predicted later chamber preference, with fish spending significantly more time on the side they initially entered (Spearman's *ρ* = 0.91, *P* < 0.001).

**Figure 3:**
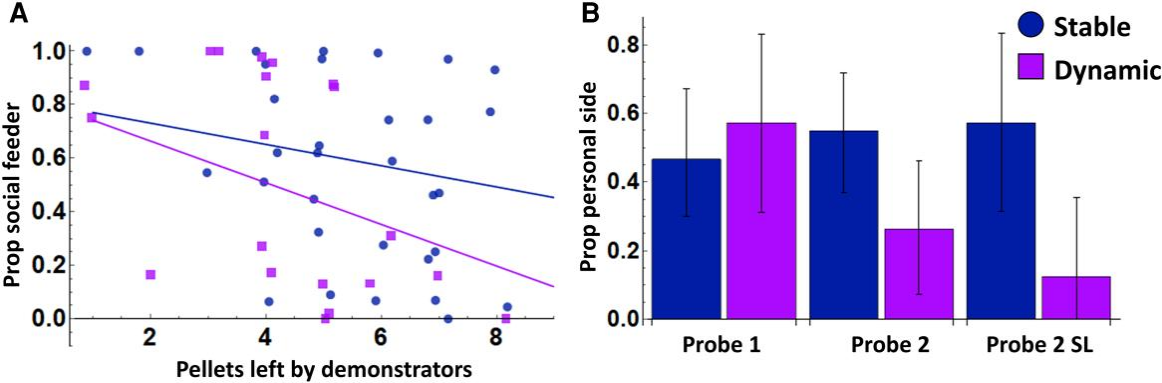
Effects of housing condition on the weighting of social information. a) scatter plot showing the relationship between demonstrator performance (number of uneaten food pellets) and test fish preference for the demonstrated feeder in the SIT; lines show linear regressions on each group's data and data points have been jittered along the *x*-axis for clarity. b) Bar chart showing the proportion of fish in the CIT that chose the personal information side first in Probe 1 (retention test), Probe 2 (after exposure to conflicting social information), and in Probe 2 restricted to successful learners (SL, fish that chose personal information in Probe 1).

Demonstrator performance predicted preference for the social information feeder, with fish spending more time near it when demonstrators had consumed a greater proportion of the food pellets (Fig. 3a; *β* = −0.05 ± 0.02, *t*(58) = −2.30, *P* = 0.025), with a significant correlation in Dynamic condition fish (*ρ* = −0.44, *P* = 0.041) and a weaker, nonsignificant trend in Stable condition fish (*ρ* = −0.30, *P* = 0.078). When restricting the data to subjects whose demonstrators ate at least half of the pellets (5 or more), fish showed a significant preference for the social information side (Wilcoxon signed-rank test, *W* = 479, *P* = 0.011), but no difference between conditions (*t*(36) = 1.06, *P* = 0.298).

A potential confound arises from demonstrators consuming significantly fewer pellets in Stable condition trials compared with Dynamic condition trials (*t*(59) = 3.47, *P* < 0.001). Dynamic condition fish also displayed a significant right-side bias, spending significantly more time in the right chamber regardless of where food had been delivered (*W* = 248, *P* = 0.02), though Stable condition fish did not (*W* = 386, *P* = 0.414). Demonstrator food consumption did not depend on the side either (*t*(59) = 0.33, *P* = 0.744).

### Conflicting information test

This assay tested how fish balanced personal and social information. Probe 1 measured first choice after 1 wk of individual training on one feeder, to assess whether fish retained personal information. Immediately after Probe 1, fish observed a demonstration of conflicting social information, where demonstrators fed at the opposite feeder. Test fish were then released into the empty arena for Probe 2, for which we measured first choice, choice latency, and time spent near each feeder, to assess whether fish conformed to social information that conflicted with their personal information. In Probe 1 (the retention test), 7 fish (5 Dynamic, 2 Stable) were removed after not making a choice for 3 min. Of the remaining fish, 22/43 (51.2%) showed retention of personal information by swimming directly to the side on which they had been trained (we term these “successful learners”: Stable 14/29, Dynamic 8/14), with no difference between conditions (χ^2^ = 0.30, *P* = 0.586). Dynamic condition fish took significantly longer to choose a chamber once released from the start box (*t*(48) = 2.36, *P* = 0.022).

In Probe 2 (post-demonstration), Dynamic condition fish were more likely to conform to social information than Stable condition fish, making an initial choice that conflicted with their prior training (Fig. 3b; Table 1; χ^2^ = 3.89, *P* = 0.049). Dynamic condition fish also chose the social information side significantly above chance (χ^2^(1) = 4.26, *P* = 0.039), whereas Stable condition fish did not (χ^2^(1) = 0.29, *P* = 0.59). Fish that conformed to social information tended to choose a side faster than those that relied on their personal information (*t*(32.39) = −2.21, *P* = 0.034), though choice speed did not differ between conditions (*t*(48) = 0.17, *P* = 0.864). This might be because decisions based on recently acquired information are made rapidly, whereas recalling older information and balancing conflicting sources requires more time. The number of leftover demonstration pellets was not related to preference (*β* = −0.12, *P* = 0.558), possibly because demonstrator foraging success was better overall than in the previous test.

Among the 22 fish that chose the trained side in Probe 1 (“successful learners”), indicating that they had learned the location of the rewarded feeder, Dynamic condition fish were significantly more likely than Stable condition fish to switch choices in Probe 2, conforming to the conflicting social information (Fig. 3b; χ^2^ = 4.13, *P* = 0.042). Dynamic condition fish chose the social information side more than chance (χ^2^(1) = 4.50, *P* = 0.034), whereas Stable condition fish did not (χ^2^(1) = 0.29, *P* = 0.593).

## Discussion

We examined how 3 mo of living under extreme environmental unpredictability (Dynamic condition) or predictability (Stable condition) affected individual behavior, collective movement, and information use in zebrafish. In all our assays, the information provided to fish did not differ across these conditions. In other words, the current experiment examines how relative differences in background environmental variability affect decision-making while controlling for any differences in the reliability of information.

Across most assays, Dynamic condition fish consistently showed greater attentiveness to and reliance on recent or current social information, higher social vigilance (paying more attention to conspecifics), and more information-seeking behavior. This was evident in their increased environmental sampling (performing more zone switches in the NTDT) and closer spacing in shoals (in the Schooling Test). However, the most direct evidence came from our explicit tests of social information use (SIT) and its relative weighting with conflicting personal information (CIT). Though fish from both conditions showed a preference for the socially demonstrated feeder when the demonstrators had consumed at least half the pellets (in the SIT), Dynamic condition fish displayed a stronger sensitivity to the quality of this social information, relying on it more when the demonstrators had consumed more of the pellets. The slope of this relationship was steeper (and only significant) in the Dynamic condition. Dynamic condition fish also showed stronger reliance on recent social information that conflicted with their personal information (in the CIT). Dynamic condition fish were the only group that significantly conformed in this test, and this effect was stronger when we only considered those fish that passed the personal information probe test. The SIT and CIT were conducted only after subjects had been exposed to their assigned environmental condition, so no comparison to pre-exposure measures was possible for these assays.

In the SIT, subjects were exposed to demonstrators either feeding or not, a form of social information, and then given a choice between the two feeders. Fish could learn by exploiting public information, in which they assess the success or failure of others to infer the quality of environmental resources (Valone 1989). However, exploiting public information is cognitively demanding, and not all species can easily detect or use it (eg, Valone and Giraldeau 1993; Smith et al. 2001; Coolen et al. 2003); there are no published data on this issue in zebrafish. Although this test provided the opportunity for public information use, zebrafish may have relied on simpler forms of social information use to solve our task, such as local enhancement (attraction to the more active group) or stimulus enhancement (attraction to the rewarded feeding ring; Hoppitt and Laland 2013).

In the CIT, fish were given a retention test (Probe 1) the day after the last day of training. Only about half of our fish showed reliable retention of the trained side, and the proportion of “successful learners” did not differ by condition. After observing a demonstration of conflicting social information (Probe 2), Dynamic condition fish were more likely to conform by choosing the social information side, whereas Stable condition fish did not show a clear preference. Successful learners (of personal information) conformed slightly more to the new social information, showing that this conformity was not simply due to forgetting their previously acquired personal information. Together, these two effects (Dynamic fish being more sensitive to the quality of social information in the SIT and conforming more in the CIT) suggest that simply living in an unpredictable environment for some time alters decision-making strategies, independent of the quality of the information itself. The CIT conformity test showed that Dynamic condition fish display a similar “copy when uncertain” strategy to other species when the reliability of their personal information is lowered (Rafacz and Templeton 2003; Laland 2004; Smolla et al. 2016). However, we additionally find that fish from dynamic environments shift to relying more heavily on social information (in the SIT), independent of the quality of the information itself, simply as a result of their long-term housing environment. Thus, their behavior may reflect a general “copy under uncertainty” strategy that is expressed across multiple contexts. Living in a physically and socially variable environment leads these fish to discount the reliability of (any) information faster and raise the value of the most recent information.

In the SPT, the social motivation of Stable condition fish decreased across exposure, while Dynamic condition fish maintained their social motivation. Living in socially dynamic environments has been shown to enhance the development of social skills and lead to higher reproductive success in male brown-headed cowbirds (*Molothrus ater*), possibly by allowing for a greater diversity of social experiences and learning opportunities (White et al. 2010). Such social competence may increase sociability through a positive feedback loop (Taborsky 2021). Thus, it is possible that the lack of opportunities to interact with novel conspecifics in the Stable condition may have diminished their social competence and motivation. Stable condition fish may have also been less attracted or responsive to the novel stimulus fish after living in unchanging groups for 3 mo, especially as their social environment was extremely consistent compared with that of wild zebrafish, who live in shoals that frequently change in size and membership (Lawrence 2007). It is alternatively possible that the reduction in social motivation resulted from general habituation which was masked, in Dynamic condition fish, by an increase in social vigilance (see above).

Collective movement, the characteristics of which are likely similarly determined by social motivation (Miller and Gerlai 2007) also differed between conditions. Dynamic condition shoals were found to be more cohesive (swimming closer together), but less coordinated (lower polarization) and slower. Correlations between polarization, distance and group speed have been noted before, and may partly result from the physics of schooling (ie, it is necessary to maintain greater distances when swimming faster, and fast-moving nonpolarized groups will disintegrate; Hemelrijk and Hildenbrandt 2008). In general, zebrafish exhibit 2 phases of collective movement, sometimes referred to as schooling (fast, polarized movement) and shoaling (slow, uncoordinated movement; Miller and Gerlai 2012a). Our results suggest that Dynamic condition groups engaged in shoaling more, and in schooling less, than Stable condition groups. The lower polarization and higher cohesion we observed in Dynamic condition shoals is consistent with our predictions that unpredictable environments should increase social vigilance and information updating, though familiarity effects may have also played a role, as discussed below. Dynamic condition fish may have oriented themselves towards one another more often, at shorter distances, to more closely monitor their less-familiar shoal-mates. Monitoring the behavior of other group members allows for faster and more accurate assessments of the environment (Valone and Templeton 2002).

However, coordinated schooling is beneficial as it can reduce energetic costs through hydrodynamic gains (Weihs 1973; Marras et al. 2015; Heydari et al. 2024; Zhang and Lauder 2024) and coordinated schools may be targeted less often by predators than weakly polarized groups (Ioannou et al. 2012). Differences in coordination between the conditions could have been a result of familiarity, as the Dynamic condition fish were less-familiar with their current tankmates (though not entirely unfamiliar, as they interacted in buckets on Switching days and had likely been housed together at some point). Familiarization decreases signaling effort in fish (Riley et al. 2019) and may increase responsiveness to signals (Thompson and Hare 2010; Micheletta et al. 2012; Carter and Wilkinson 2016). However, the empirical evidence for better schooling in familiar groups is mixed. In tropical damselfish (*Chromis viridis*), there is no difference in cohesion or coordination between familiar and unfamiliar shoals (though familiarity improves escape performance; Nadler et al. 2021). In female guppies (*Poecilia reticulata*), familiarity increases polarization but has no effect on cohesion (Davis et al. 2017). Fathead minnows (*Pimephales promelas*) and Mediterranean killifish (*Aphanius fasciatus*) both show *greater* cohesion in familiar shoals, the opposite of our results (Mathis and Smith 1993; Lucon-Xiccato et al. 2022).

Stable condition fish may also have swum further apart from each other as a result of competitive strategies. While grouping has benefits, such as protection from predators (Ward and Webster 2016), it also entails costs, including competition among group members (Brockmann and Barnard 1979; Ward and Webster 2016). Such costs can outweigh the benefits, reducing sociality in species that evolved in resource-scarce environments (Shier and Randall 2004; Sekulovski and Miller 2025). For example, zebrafish have been shown to attempt to avoid kleptoparasitism when foraging in the presence of others (Sekulovski et al. 2026) and, when food delivery is predictable in time and space (as in our Stable condition), will attempt to monopolize the food source (Hamilton and Dill 2002). Similar competitive strategies and aggression have been shown to arise in other species (eg, Robb and Grant 1998; Goldberg et al. 2001; Kleiber et al. 2022). It is possible that prolonged predictable food delivery in the Stable group, at a level of consistency unusual in the wild, shifted their behavior towards competitive rather than cooperative strategies, further amplifying the difference in attitudes to social stimuli between the two conditions.

Exploratory behavior (time spent outside of shelter in the OFT) increased and stress (time spent freezing in the NTDT) decreased after exposure in both conditions, suggesting that fish habituated to the testing tanks or, more generally, to frequent handling and being placed in novel arenas. Exploration is positively correlated with boldness in zebrafish (dos Santos et al. 2023), and fish tend to become bolder in captivity (Huntingford 2004; Huntingford and Adams 2005; Agnvall et al. 2015), likely due to the lower costs of risk-taking in such environments. For example, in laboratory settings, bolder and more aggressive fish have more reproductive and foraging success as there are no predation risks (Ward et al. 2004; Ariyomo and Watt 2012). Fish in our study did not differ in the amount of time they spent near the bottom of the tank or freezing in the NTDT, nor in their tendency to stay near the walls while swimming in the Schooling Test, suggesting that the differences observed in the other assays are unlikely to be a result of heightened stress in one condition. However, the Dynamic condition had a higher mortality rate throughout the experiment. While the cause of this mortality is unknown, and no overt signs of disease were observed, it may have involved stress-related immunocompromise, as reported in other fish species exposed to fluctuating water temperatures (Franke et al. 2024). Fish in the Dynamic condition were also exposed to social mixing, which is known to also increase the risk of disease transmission, a major cost of group living and possibly related to the reason many fish species prefer to school with familiar individuals (Croft et al. 2005).

Our findings provide empirical evidence that environmental unpredictability increases the value of recent information and promotes information-seeking behavior, which may reflect a greater motivation to resolve uncertainty (Dall et al. 2005; Bennett et al. 2016; Dridi and Lehmann 2016). They are also in line with existing studies that show that the quality of information shifts decision-making strategies: when personal information is less reliable, animals shift to using social information (Bell and Baum 2002; van Bergen et al. 2004; Kendal et al. 2009). Our study adds that simply living in a highly variable environment is sufficient to change the perceived quality of information, degrading information faster and shifting to the “copy when uncertain” strategy, independent of the actual quality of available information (Bell and Baum 2002; van Bergen et al. 2004; Kendal et al. 2009). This result supports theoretical models in which environmental change reduces the predictive value of older information and favors recent cues (Boyd and Richerson 1988).

We also found that fish from unpredictable environments find social information more salient than fish in the Stable condition. The increased attention to and reliance on social information may be due to it being more easily and cheaply acquired and updated—keeping up with changing conditions—making it more reliable in variable environments than personal information (Laland 2004; Kendal et al. 2009). Fish from both conditions tended to use more reliable (recent) information, but Dynamic condition fish were more likely to follow the most accurate social information and less likely to follow less reliable (older) or unclear information, possibly because information can become outdated more quickly and mistakes may entail more severe costs in unpredictable environments. Under stable conditions, information changes less. Animals may therefore develop a weaker sensitivity to uncertainty and devote less attention to environmental cues, such as social stimuli. Because less updating is required, information-seeking declines (Dall et al. 2005; Dridi and Lehmann 2016). Additionally, living in a stable social group reduces the need to monitor and track group members and social dynamics (Amici et al. 2008; Aureli et al. 2008; MacLean et al. 2012), and the predictability of the group's composition may lower the salience of social stimuli, because familiar individuals provide less novel information. This reduced salience of social stimuli may also translate into lower responsiveness to unfamiliar conspecifics, as we observed in the SPT.

Importantly, our Stable condition did not constitute a neutral control, but rather an unusually predictable environment for a species that evolved in highly variable habitats and inhabits large fission-fusion shoals (Daniels 2002; Bhat 2003; Engeszer et al. 2007; Lawrence 2007; Spence et al. 2008; Shelton et al. 2020). From this perspective, the Dynamic condition may have provided a more naturalistic environment, with more realistic social learning opportunities. Accordingly, some of the behavioral differences we observed between conditions may be due to the unusually consistent information available to fish in the Stable condition, providing fewer opportunities for updating through learning.

Dynamic environments simulated in the lab may capture some aspects of the variability animals experience in the wild, providing insight into how they might respond to intensifying global environmental change. Because we applied both social and physical manipulations, the behavioral differences we observed between conditions could have arisen from fluctuations in the social environment (such as group composition or size), physical environment (such as water temperature, feeding times, or habitat complexity), or from a combination of both. Future research could test these environmental variables separately to reveal which factors drove the observed effects. In addition, because fish were not individually identifiable, our analyses were limited to the group level, and pre and post-exposure comparisons could not be made for individual fish. This limited our ability to determine the extent to which the observed condition differences were due to behavioral changes within individuals or group-level effects. Future research could include individual identification, such as tagging, to examine how individual differences shape behavioral responses to environmental unpredictability.

Although we did not directly measure aggression, social instability (as experienced by fish in our Dynamic condition) might increase conflict by repeatedly disrupting social hierarchies. Frequent group-membership changes elevate social uncertainty and may temporarily increase aggression until dominance hierarchies re-stabilize (Utne-Palm and Hart 2000; Larson et al. 2006). For example, zebrafish often take up to 5 d to stabilize dominant-subordinate relationships, after which aggression decreases (Larson et al. 2006). In contrast, predictable food delivery may also increase aggression as dominant fish attempt to monopolize resources (eg, Goldberg et al. 2001; Hamilton and Dill 2002). Although no differences in aggression were observed between the conditions during the behavioral assays, future studies could include explicit measures of aggression. Future research could also test whether environmental predictability has developmental (organizational) effects by exposing subjects to stable versus dynamic conditions earlier in their lives. This was not possible in the current study, as we wished to equalize the initial mean personality trait values and stress levels across conditions, which required testing the adult fish before assignment to housing conditions.

Overall, our study shows that environmental instability shifts priority toward recent information, heightens information-seeking behavior, and disrupts schooling by reducing group coordination. These findings may have important implications for how animals will alter their behavior and decision-making strategies in an increasingly unpredictable world.

## Supplementary Material

arag064_Supplementary_Data

## Data Availability

Analyses reported in this article can be reproduced using the data provided by Sekulovski and Miller (2026).
