# Supplementary material for: Long-term environmental unpredictability increases social information use in zebrafish
Source: Behav Ecol. 2026 Jun 10;37(4):arag064. doi: 10.1093/beheco/arag064 (PMC13270242; doi:10.1093/beheco/arag064)

**Supplementary Materials**

**Supplementary methods**

**Open-Field Test (OFT)**

*Apparatus*: Exploratory behaviour was measured in a Styrofoam tank (57 cm × 36 cm × 8 cm water depth; Figure S1), with a sheltered area (a strip of white corrugated plastic, along one short wall at the water surface; 36 cm × 8 cm) containing artificial plants glued along the underside. LED lights were placed next to the tank and a white shower curtain surrounded it. An overhead webcam (Logitech C920) recorded the OFT and the adjacent SPT simultaneously.

*Procedure*: The tank was filled with water matched to home tanks, refreshed daily. Each fish was net-transferred to the center of the tank and a 15-min trial began after the curtain was closed. The order of OFT and SPT was counterbalanced across fish. Each fish was tested once before housing condition assignment and once immediately after the 3-month exposure.

*Tracking and Analysis*: A custom Python script tracked fish in real time at 30 fps, extracting X-Y coordinates for every frame and calculating the total proportion of time fish spent outside of the shelter zone.

**Social-Preference Test (SPT)**

*Apparatus*: Social motivation was measured using a three-chamber glass tank (75 cm × 29.5 cm × 9 cm water depth; Figure S1), divided by clear plastic partitions into a 50.5 cm central test chamber and two 12 cm side stimulus chambers. The interior was lined with opaque white waterproof paper. See OFT for surroundings.

*Procedure*: The tank was filled with water taken from home tanks, refreshed daily. One stimulus chamber held five novel fish, while the other stimulus chamber remained empty. The side on which the stimulus shoal appeared was counterbalanced across subjects. Stimulus fish were placed in the tank at least 5 min before testing. Each fish was net-transferred to the center of the tank and a 15-min trial began after the curtain was closed. Each fish was tested once before housing condition assignment and once immediately after the 3-month exposure.

*Tracking and Analysis*: Real-time tracking was used to compute the total proportion of time fish spent in close proximity (within 10 cm) to the shoal chamber.

**Novel-Tank Diving Test (NTDT)**

*Apparatus*: Stress reactivity was evaluated using an established NTDT (Egan et al., 2009; Levin et al., 2007) in right trapezoid 5 L clear plastic tanks (32.5 cm top /26 cm bottom × 10.5 cm × 15 cm water depth; Figure S2). Two tanks were on a table and two were elevated on a riser directly above, allowing four fish to be recorded simultaneously. Opaque white PVC walls behind the tanks enhanced contrast for side-view tracking and walls between adjacent tanks provided visual isolation. The front faces of the tanks were marked externally into equal-depth bottom, middle, and top thirds (5 cm per zone). LED bars on the sides illuminated the tanks, and a tripod-mounted webcam (Logitech C920; 30 fps) recorded the side-view of all four tanks.

*Procedure*: Tanks were filled with system water matched to home tanks, refreshed daily. Fish were individually net-transferred to one of the tanks and allowed ~10 s to settle and a 10 min trial began once the room door closed. Each fish completed the assay twice: Pre-exposure and Post-exposure. Pre-exposure NTDT trials were run across five days, directly after the OFT and SPT. Post-exposure trials were completed five days after the Post-exposure OFT and SPT.

*Tracking & Analysis:* A custom Python script generated X-Y coordinates per frame. Dependent variables were time spent in bottom and top thirds (middle excluded), freezing duration, and vertical zone switches. A greater proportion of time in the bottom third and increased freezing were interpreted as heightened stress.

**Schooling Test**

*Apparatus*: Group movement was measured in a white round rubber tub (60 cm diameter; 10 cm water depth; Figure S3). Overhead recording (Canon Vixia HF R700; 30 fps) captured behaviour.

*Procedure*: The tub was filled with system water matched to home tanks, refreshed after every third trial. Five fish from the same condition and (current) tank were net-transferred to the center of the tub and allowed to swim freely for 10 min. All fish completed this test within a week after completing the NTDT. Fish received this test and the follow Post-exposure only.

*Tracking & Analysis*: A custom Python script tracked fish positions and a custom R script calculated inter-individual distance (IID), nearest-neighbour distance (NND), polarization, speed, and thigmotaxis. The IID is the mean distance between each fish and every other fish, averaged over all focal fish; the NND is the mean distance between each fish and the closest fish to it; polarization is the degree to which all fish are swimming in the same direction (and varies from 0 to 1); and thigmotaxis is tendency to stay close to the wall (within the outer 10% of the tank radius). Metrics were averaged over the full session and within four consecutive 2.5 min epochs to examine changes across time.

**Social Information Test (SIT)**

*Apparatus*: Social information use was tested using a modified demonstrator-observer paradigm (Coolen et al., 2003; van Bergen et al., 2004; Webster & Laland, 2011), in a white PVC arena (99.5 cm × 59 cm, 12 cm water depth; Figure S4), with a triangular divider partially split the tank (43 × 37 cm), creating two feeding chambers (28 top × 49 bottom × 37 cm) and a long front neutral zone (99.5 × 22 cm). Each feeding chamber contained a floating red plastic ring feeder (diameter: 6 cm) positioned beneath an externally mounted acrylic tube for pellet delivery. A transparent half-cylinder start box (6.5 × 4.5 × 28.5 cm), with a nylon pull line, was placed midway along a long wall. Removable barriers (one transparent barrier 98 × 25 cm; two shorter opaque black barriers 47 × 20.5 cm) were used to alternately enclose or reveal the feeding chambers. The white shower curtains covered the sides of the arena, and an overhead webcam (Logitech C920) recorded probe trials.

*Procedure*: The arena was filled with system water matched to home tanks and refilled every third day, with heaters and airstones run overnight to maintain temperature and oxygenation.

To simplify the typical SIT, demonstrators were presented with a fed vs. unfed feeder, rather than a rich vs. poor feeder (i.e., more food vs. less food; Coolen et al., 2003; van Bergen et al., 2004; Webster & Laland, 2011). Approximately 30 demonstrator fish were pre-selected for the SIT and CIT based on whether they would feed in a novel environment to ensure more consistent demonstrator performance. Demonstrators were fed exclusively on test food pellets used during testing (Hikari Betta Bio Gold; 1 mm diameter) for at least 7 days prior to training (and continued after the SIT until the CIT). Over two consecutive days before testing, demonstrators were placed in the arena for 20 min with food delivered to both feeders and only fed in the arena.

Test fish were fasted for 24 h before testing and were habituated to the arena for 20 min in their tank groups, with all barriers removed the day before testing. For the SIT, with all barriers closed, three demonstrators were net-transferred to each feeding chamber. Demonstrators swapped chambers between trials and only participated in one or two (never consecutively) feeding demonstrations per day to maintain consistent feeding motivation. Test fish were individually poured into the start box with a beaker, acclimating for 2 min before opaque barriers were removed, leaving the transparent barrier. After 30 s, ten food pellets were delivered to one ring feeder (sides counterbalanced across subjects) via the attached tubes (ensuring that the experimenter was not visible to the fish). Demonstrators were allowed to feed for 8 min while the test fish observed. Following the demonstration, opaque barriers were reinserted, and demonstrators and pellets, if any, were removed. Uneaten pellets were counted and reported. Water was briefly cross-mixed between feeding chambers using a beaker to control for residual olfactory cues. All barriers were then removed and, after a 30 s delay, the test fish was released into the arena for 5 min.

*Tracking & Analysis*: Videos were manually tracked using a custom Python script, extracting the frame number each time the fish entered a zone. ROIs included the neural zone, each feeding chamber, and around each ring feeder. The variables of interest were the proportion of time the fish spent on each side of the arena, initial choice, and the number of pellets left uneaten by the demonstrators.

**Conflicting Information Test (CIT)**

*Apparatus*: The CIT used the same arena as the SIT (Figure S4).

*Procedure*: Fish completed the CIT within approximately two months after the SIT. Test fish were exclusively fed the test pellets for at least seven days before training (~3 pellets per fish per feeding), using ring feeders placed in the home tanks and fasted for 24 h before the first day of personal information. The arena was filled with system water matched to home tanks and refilled every third day, with heaters and airstones run overnight to maintain temperature and oxygenation.

Fish were trained in groups (current tankmates) for seven consecutive days to associate one side of the arena with food. With only the transparent barrier in place, each tank was split randomly between the two feeding chambers, and after 30 s, 20 pellets were delivered to the ring feeder on one designated side (counterbalanced across tanks and conditions) and never the other (i.e., only half the fish were fed). Fish fed for 15 min before being net-transferred to the opposite chamber for the next trial. Each tank received four training trials per day (two per side) and only fed during training on these seven days. On the final day of personal information training, all barriers were removed and test fish were placed in the arena overnight in their home tank groups for habituation to decrease exploratory behaviour during testing. Over two consecutive days before testing, demonstrators were placed in the arena for 20 min with food delivered to both feeders, always after test fish completed their training.

Each fish was tested individually on their learned side preference (Probe 1) the day after the last day of training. All barriers were removed from the arena, and the test fish was poured into the start box. After 1 min of acclimation, the fish was released, and then removed immediately following their first choice (crossing fully into one chamber). If a fish did not make a choice within 3 min, it was removed and excluded from analysis. All fish then proceeded directly to the public demonstration, regardless of choice. The public demonstration and Probe 2 were identical to the SIT, except demonstrators fed on the opposite side from the one associated with food during personal training, creating conflict between privately learned and publicly observed information about the location of the food.

*Tracking & Analysis*: As in SIT, manual Python tracking extracted time spent in each zone. The same variables of interest from the SIT, as well as the latency to make an initial choice during both Probe 1 and 2, the number of switches, and whether fish shifted their preference toward the demonstrated location (indicating conformity; van Bergen et al., 2004) were examined.

**Table S1.**

| Day | Temp (°C) | Feeding | Group N | Plants |
| --- | --- | --- | --- | --- |
| 1 | 20 | 10, 14 | 10, 10, 10, 10 | 2 |
| 2 | 22 | 11, 13 |  |  |
| 3 | 26 | 10, 16 |  |  |
| 4 | 27 | 12, 15 | 5, 5, 15, 15 | 7 |
| 5 | 24 | 10, 15 |  |  |
| 6 | 19 | 12, 16 |  |  |
| 7 | 18 | 10, 12 | 9, 9, 11, 11 | 5 |
| 8 | 21 | 10, 16 |  |  |
| 9 | 25 | 11, 14 |  |  |
| 10 | 28 | 11, 16 | 8, 8, 12, 12 | 1 |
| 11 | 26 | 10, 14 |  |  |
| 12 | 22 | 12, 14 |  |  |
| 13 | 27 | 10, 16 | 6, 6, 14, 14 | 3 |
| 14 | 25 | 13, 16 |  |  |
| 15 | 20 | 10, 15 |  |  |
| 16 | 19 | 11, 15 | 7, 7, 13, 13 | 6 |
| 17 | 21 | 14, 16 |  |  |
| 18 | 24 | 12, 14 |  |  |

Dynamic condition environmental variation. In Dynamic condition tanks, the environment was varied following this table, on a repeating 18-day cycle. Water temperature (**Temp**) varied each day, with no more than a 5 °C change per day. All values were within ranges that zebrafish can easily tolerate (Lawrence, 2007), and the mean value across the cycle matched the temperature maintained in the Stable condition tanks. Feeding times (**Feeding**) also varied daily, pseudo-randomly between 10:00 and 16:00 h, with a minimum of 2 h between feedings. Mean feeding times matched those in the Stable tanks. Group size & composition (**Group N**) was varied every third day (on Switching days). On these days, all 40 Dynamic condition fish were net-transferred into one bucket and then randomly redistributed across the four tanks in pseudorandom group sizes (5-15 fish per tank). Mean group size across the cycle was 10 fish per tank, matching the Stable tanks. Finally, habitat complexity (**Plants**) was varied by changing the number of artificial plants in the tank on Switching days. The mean across the cycle was 4 plants, as in the Stable tanks.

**Figure S1**: Open-Field Test and Social-Preference Test. Diagram (left) and photo (right) of the open-field test arena (on the left of each pair) and the social-preference test arena (on the right). The black area indicates a shelter; dotted lines indicate transparent walls. In the photos, which are screenshots from the automated tracking program, green lines show the tracking ROI and the test fish are outlined in red.

**
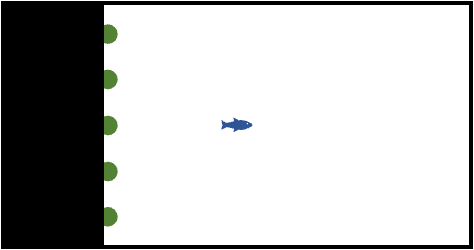

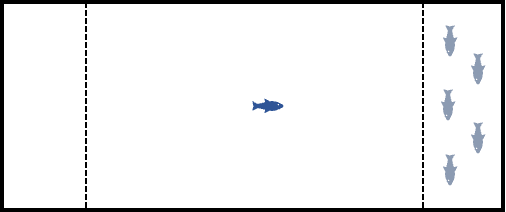

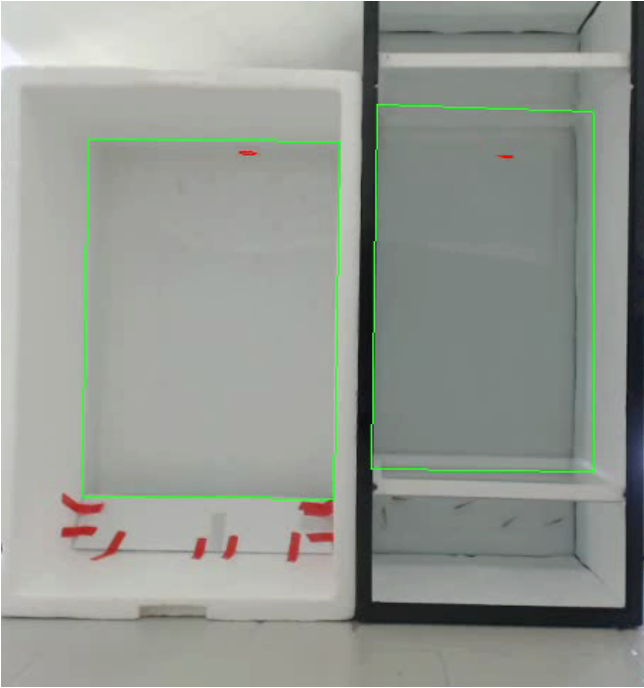
**

**Figure S2**: Novel-Tank Diving Test. Diagram (left) and photo (right) of the novel-tank diving test arena. Dashed lines show the limits of the three vertical zones, used for analysis. In the photo, which is a screenshot from the automated tracking program, green lines show the tracking ROIs for each tank, and the test fish are outlined in red.

**
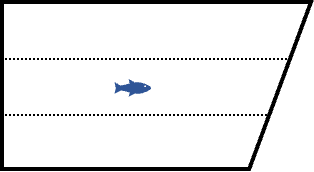

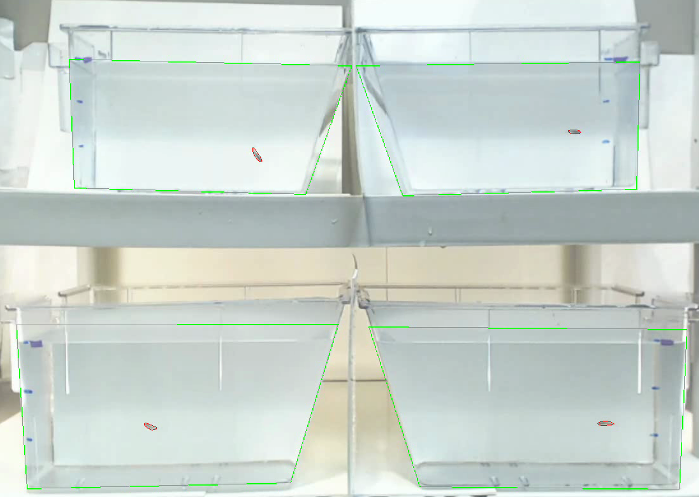
**

**Figure S3**: Schooling Test. Diagram (left) and photo (right) of the schooling test arena. In the photo, which is a screenshot from the automated tracking program,fish are indicated by colored and labeled dots.

**
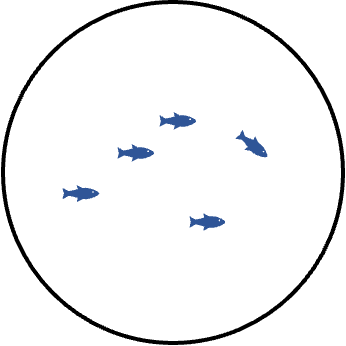

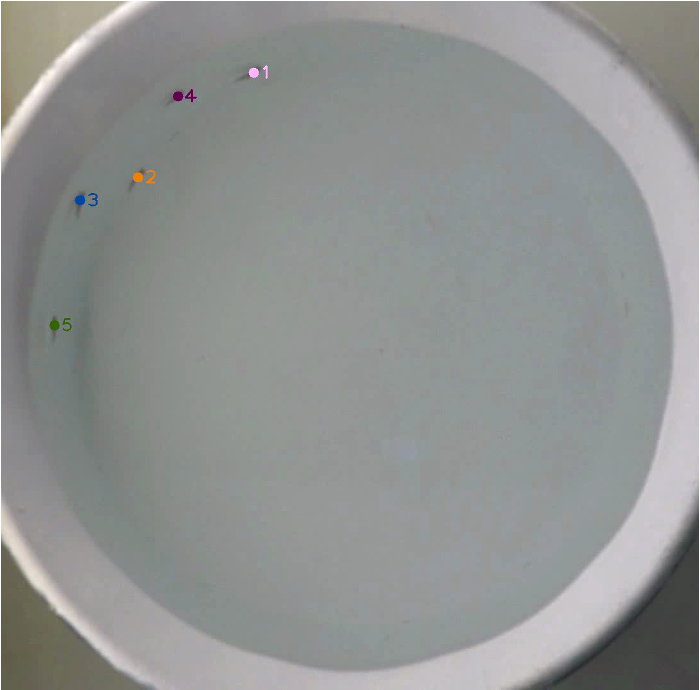
**

**Figure S4**: Social Information Test and Conflicting Information Test (during public demonstration). Diagram (top) and photo (bottom) of the arena used for both the public information test and the conflicting information test. Dashed black lines indicate a transparent wall. Red dashed lines indicate the tracking zones. Blue circles are feeders.

**
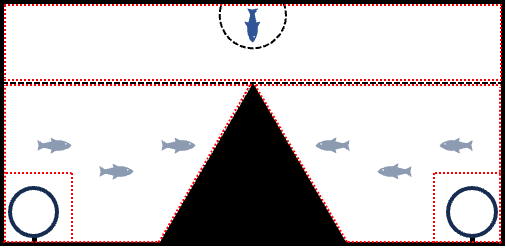
**

**
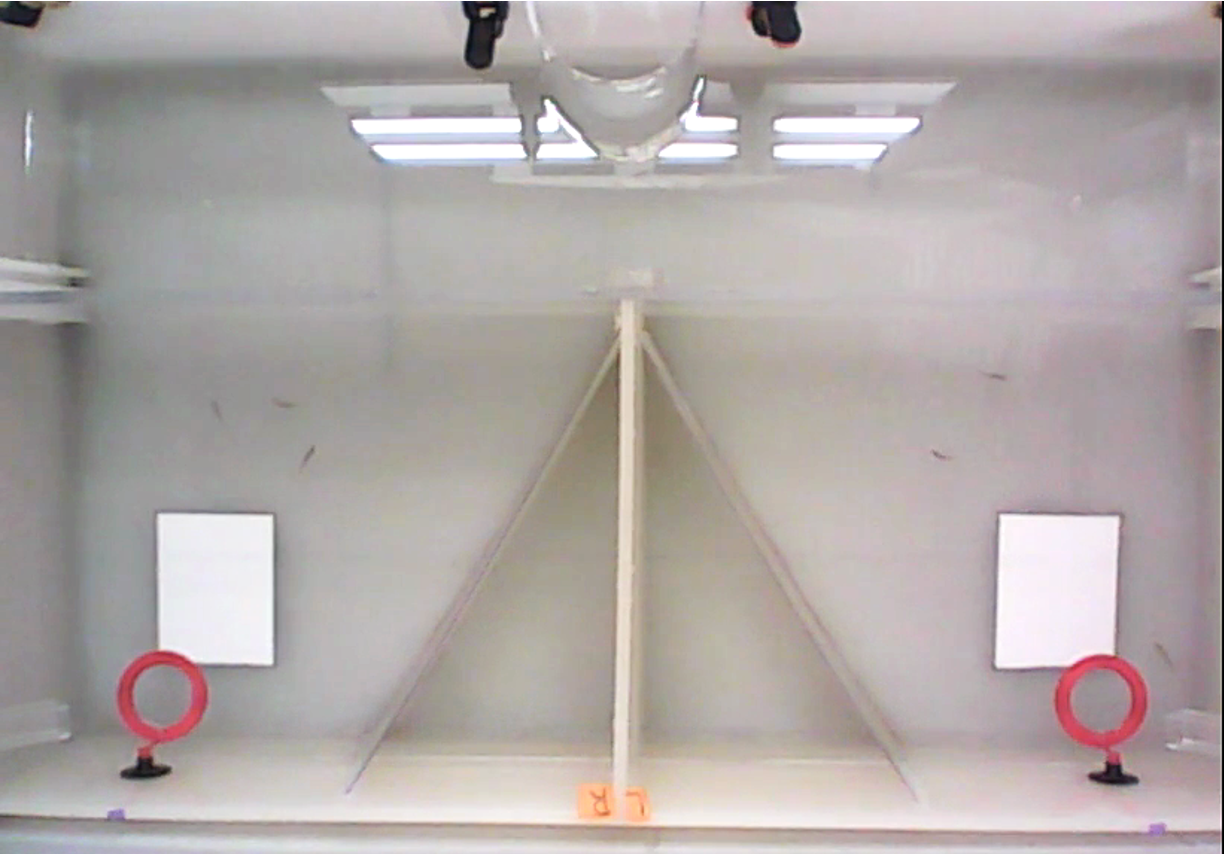
**

**Figure S5.** Effects of housing condition on stress reactivity. Violin plots of measures from the Novel Tank Diving Test (NTDT) for the Stable (“St”, blue shades) and Dynamic (“Dy”, purple shades) conditions both before (“Pre-exp”, light shades) and after (“Post-exp”, dark shades) a 3-month exposure to varying housing conditions. A: proportion of time spent in the bottom third of the tank during the entire 10-min session; B: proportion of time in the bottom third during the first half of the session; C: proportion of time spent freezing; D: number of vertical zone crossings. Short horizontal black lines show group means. Colours are as in Figure 1 in the main paper.


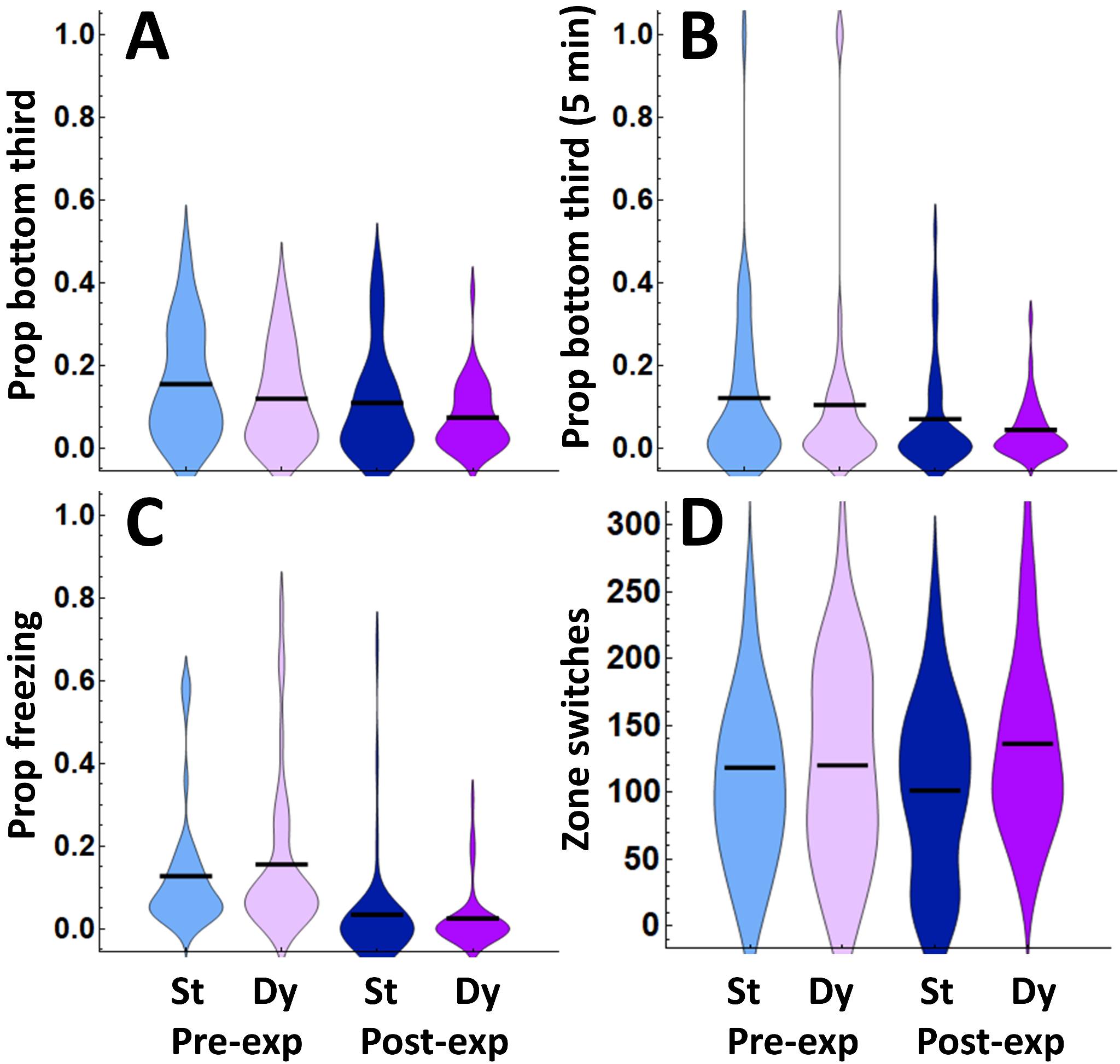

Supplement: arag064_Supplementary_Data [file arag064_supplementary_data.docx]
